# Lipid Profile in Tuberculosis Patients with and without Human Immunodeficiency Virus Infection

**DOI:** 10.1155/2017/3843291

**Published:** 2017-11-01

**Authors:** Gebremedhin Gebremicael, Yemane Amare, Feyissa Challa, Atsbeha Gebreegziabxier, Girmay Medhin, Mistire Wolde, Desta Kassa

**Affiliations:** ^1^HIV and TB Research Directorate, Ethiopian Public Health Institute (EPHI), Addis Ababa, Ethiopia; ^2^Institute of Biomedical Science, College of Health Sciences, Mekelle University, Mekele, Ethiopia; ^3^Aklilu Lemma Institute of Pathobiology, Addis Ababa University, Addis Ababa, Ethiopia; ^4^Department of Medical Laboratory Sciences, Addis Ababa University, Addis Ababa, Ethiopia

## Abstract

**Background:**

Understanding whether the preceding low lipid profile leads to active tuberculosis (TB) or active TB leads to low lipid profile is crucial.

**Methods:**

Lipid profile concentrations were determined from 159 study participants composed of 93 active TB patients [44 HIV coinfected (HIV+TB+) and 49 HIV negative (HIV−TB+)], 41 tuberculin skin test (TST) positive cases [17 HIV coinfected (HIV+TST+) and 24 HIV negative (HIV−TST+)], and 25 healthy controls (HIV−TST−). Cobas Integra 400 Plus was used to determine lipid profiles concentration level.

**Results:**

The concentrations of total cholesterol (TC), triglyceride (TG), low-density lipoprotein cholesterol (LDL-C), and high-density lipoprotein cholesterol (HDL-C) in HIV−TB+ patients were significantly lower compared to HIV−TST+ and to HIV−TST− individuals. Similarly, the concentrations of the TC, LDL-C, and HDL-C in HIV+TB+ were significantly lower compared to HIV−TB+ patients. After the 6 months of anti-TB treatment (ATT), the concentration levels of TC, LDL-C, and HDL-C in HIV−TB+ patients were higher compared to the baseline concentration levels, while they were not significantly different compared to that of HIV−TST+ concentration.

**Conclusion:**

The low concentration of lipid profiles in TB patients may be a consequence of the disease and significantly increased in TB patients after treatment.

## 1. Introduction

Cholesterol regulates hormones in the body and basic cellular metabolism. LDL and HDL transport cholesterol back and forth between tissues and liver [1]. Cholesterol is used to make fluidic of cell membrane structure, participating in the activity of enzymes and in the functions of phagocytosis and cell growth [2, 3]. Accumulation of cholesterol in the blood vessels narrows the blood tube and causes atherosclerosis [4].

Current recommendations for the treatment of noncommunicable diseases are all geared towards reducing the serum cholesterol levels [5]. However, increasing evidence indicates a link between low blood cholesterol levels and a number of human diseases including tuberculosis (TB) [6]. TB has remained a major global health threat for centuries [7]. About 90–95% of immune-competent latent TB infected persons will not develop TB disease in their lives. Nevertheless, when the immune systems become weak, especially those with HIV infection patients, the developing TB diseases are considerably higher [8]. Hypocholesterolemia promotes the development of TB whereas hypercholesterolemia leads to protection against TB with* Mycobacterium tuberculosis* (Mtb) [9].

Cholesterol in the lymphocyte cell membrane is important for their cytotoxic function [10]. Activated lymphocyte subsets including CD4+, CD8+, and T cells recruit macrophages and release molecules, such as interferon and tumour necrosis factor that render them more efficient in killing Mtb [11]. The inability of macrophages to uptake Mtb due to a low cholesterol content of their cell membrane might constitute a key defect in the host defence system against TB [11]. The level and function of lipids profile were negatively affected by pulmonary TB [12]. TB makes malnutrition worse, and malnutrition weakens immunity, thereby increasing the likelihood that latent TB will develop into active disease [13]. But it is not clear whether the preceding low lipid profile leads to an advancement of the disease or active TB leads to low lipid profile. In Ethiopia, there is not enough data on lipid profiles level in TB patients with and without HIV infection, though there is a study done by Tadewos et al. [14] which assessed the prevalence of dyslipidemia among HIV-infected patients on first-line HAART in Southern Ethiopia. Therefore, the aim of this study was to determine any differences in lipid profile between TB patients with and without HIV infection and controls (latent TB and healthy controls) and to determine the level of lipid profiles in response to TB treatment.

## 2. Methods

### 2.1. Study Designs and Populations

An observational study was conducted at St. Peter Specialized TB Hospital, Akaki and Kality Health Centers, Addis Ababa, Ethiopia, between April 2007 and January 2011. A total of 159 adults of both sexes ranging between 15 and 65 years of age were enrolled. Study participants were composed of 93 active TB patients with newly diagnosed tuberculosis (never treated) [44 HIV coinfected (HIV+TB+) and 49 HIV negative (HIV−TB+)], 41 latent TB cases [17 HIV coinfected (HIV+TST+) and 24 HIV negative (HIV−TST+)], and 25 healthy controls (HIV−TST−). At recruitment, all study participants were interviewed using a standard questionnaire and demographic data were collected. A control group of 49 (HIV−TST+ and HIV−TST−) subjects without a prior diagnosis of TB was recruited from the group of individuals who works at Ethiopian Public Health Institute (former name Ethiopian Health and Nutrition Research Institute) as daily labourer without any clinical symptoms or signs of illness due to active TB. The blood plasma was separated from each patient's whole blood at HIV national laboratory and then stored in a deep freezer until use. Demographic data were collected at the baseline during recruitment. Exclusion criteria for enrollment were refusal of HIV testing, pregnancy, comorbidity with diabetes mellitus or chronic bronchitis, receiving steroid therapy, receiving TB and/or HAART treatment (at recruitment or previously), and alcohol or drug abuse that could compromise reliability. All active TB cases confirmed at enrollment were treated according to the national guideline [15].

### 2.2. Diagnostic Assessments

The HIV status of study participants was determined using the Determine HIV-1/2 (Abbott laboratories, Japan) as the screening test, the Capillus HIV-1/2 (Trinity Biotech, Ireland) as the confirmatory test and Uni-Gold HIV-1/2 recombinant (Trinity Biotech, Ireland) as a tie breaker test [15]. The CD4 T cell count was determined by flow cytometry using a FACSCalibur Flow cytometer (Becton Dickinson, San Jose, USA).

Active TB diagnosis was based on both clinical and bacteriological parameters. At least two sputum smears (“spot-early morning”) were required to be positive by microscopy for Acid Fast Bacilli (AFB) using the Ziehl-Neelsen staining method and X-ray positive for those participants presenting with a dry cough [15]. A TST test to detect latent TB infection was performed at baseline visits for all participants except active TB patients according to the national guidelines [15]. A 0.1 ml tuberculin solution (RT23, State Serum Institute, Copenhagen) was injected intradermally into the dorsal surface of the forearm: TST positivity was classified as skin induration diameter ≥ 5 mm in HIV-infected individuals while being ≥10 mm in HIV negative individuals [15].

### 2.3. Lipid Profiles Testing

TC, TG, HDL-C, and LDL-C were measured using stored plasma samples separated from fasting blood sample collected from a peripheral vein and Cobas Integra 400 Plus clinical chemistry machine (Roche, Switzerland). Two levels of quality control materials (PreciControl CliniChem Multi 1 and 2, Roche 05 117 208 922, 05 117 291 922) were run prior to sample testing and analysis each day to set instrument sensitivity. In addition, the Cobas Integra 400 Plus instrument at the national reference laboratory was enrolled in an external quality assurance program (EQA- One world Accuracy) to maintain good laboratory practice (GLP). The instrument passed the EQA assessment during the study and during the preceding year of EQA panels. A sample of the individuals with a result of TC < 130 mg/dl, TG < 90 mg/dl, LDL-C < 100 mg/dl, and HDL-C < 40 mg/dl were considered as hypolipidemic while individuals with the result of TC ≥ 200, TG ≥ 150, LDL-C ≥ 130 mg/dl, or HDL-C > 40 mg/dl were classified as hyperlipidemic in both groups [5].

### 2.4. Statistical Analyses

A Kruskal-Wallis test was used to compare medians between more than two clinical groups, while a two-tailed Wilcoxon rank-sum (Mann–Whitney) test was used to compare two unpaired data items or a Wilcoxon signed-rank test for two paired group data items. A Chi^2^ test was used to compare proportions of dichotomous variables across the groups. Logistic regression analysis was performed in patients and controls for factors affecting TB. All data analysis was done using STATA version 11.0 (College Station, Texas, USA). *P* value of 5% was taken as a cutoff to determine statistical significance.

## 3. Results

### 3.1. Characteristics of the Study Population at Baseline

A total of 93 TB patients [49 HIV−TB+ and 44 HIV+TB+], 41 latent TB cases [17 HIV+TST+ and 24 HIV−TST+], and 25 HIV−TST− were included in the study.  Table 1 shows the demographic, nutritional, and clinical status of the patients and controls. Malnutrition (Body Mass Index (BMI) < 18.5 kg/m2) was not detected in all study groups. There was no statistically significant difference in the median BMI of the study groups (*P* value > 0.05). The proportion of patients with the CD4-T cell of less than 200 cells/*μ*l had significant difference among the groups (*P* = 0.003), and HIV+TB+ and HIV−TB+ individuals had been with the higher proportion (Table 1).

### 3.2. Lipid Profile Status of Active and Latent TB in HIV Negative Study Groups

The concentrations of four lipid profiles (TC, TG, LDL-C, and HDL-C) were significantly lower in HIV−TB+ compared to HIV−TST+ and HIV−TST−. However, there were no statistically significant differences in concentrations of TC, TG, LDL-C, and HDL-C between HIV−TST+ and HIV−TST− (Table 2(a)).

Table 2(b) shows the proportion of hypolipidemia and hyperlipidemia of the patients and controls. The hypolipidemia of TC, TG, LDL-C, and HDL-C was significantly more prevalent in HIV−TB+ than in HIV−TST+ and HIV−TST− (*P* < 0.05). The proportion of hypolipidemia of all four lipid profiles (TC, TG, LDL-C, and HDL-C) was detected in 44% of HIV−TB+ compared to 3% of HIV−TST+ and 0% of HIV−TST− (Table 2(b)). In the bivariate logistic regression analysis, hypolipidemia of TC, TG, LDL-C, and HDL-C was associated with TB (odds ratio: 2.9, 1.75, 1.64, and 1.89, resp., *P* < 0.007). Hypolipidemia of TC, TG, LDL-C, and HDL-C was not associated with latent TB; this finding could be due to the low numbers of patients with this hypolipidemia.

### 3.3. The Effect of HIV on the Concentration of Lipid Profile in Active TB and Latent TB Study Participants

The concentrations of the three lipid profiles (TC, LDL-C, and HDL-C) were significantly lower in HIV+TB+ patients compared to HIV−TB+ patients. Moreover, the concentrations of these three lipid profiles were lower in HIV+TST+ patients compared to HIV−TST+ patients (Table 3). There was no significant difference on the concentration level of TG between HIV+TB+ and HIV−TB+ patients and between HIV+TST+ and HIV−TST+ patients (Table 3). The proportion of hypolipidemia of all four lipid profiles (TC, TG, LDL-C, and HDL-C) was detected in 40% of HIV+TB+ patients (Table 2(b)).

### 3.4. Anti-TB Treatment (ATT) Response on Lipid Profile Concentration in HIV−TB+ Patients

In this study, we assessed also the effect of ATT treatment on the level lipid profiles. Thus, the concentrations of TC, TG, LDL-C, and HDL-C in HIV−TB+ patients were measured at six months (M6) of ATT and compared to the baseline value (M0) of the same patients (HIV−TB+) and with that of apparently healthy control (HIV−TST+ and HIV−TST− groups).

The concentrations of TC, LDL-C, and HDL-C were significantly higher in HIV−TB+ patients at the end of ATT compared to HIV−TB+ patients at baseline, while the concentration of TG was not different between before and after ATT of HIV−TB+ patients (Table 4).

Although baseline concentrations of the lipids were lower in HIV−TB+ individuals compared to HIV−TST+ and HIV−TST− (Table 2(a)); the concentration of all the lipids (TC, TG, LDL-C, and HDL-C) after ATT in HIV−TB+ had no significant difference compared to that of HIV−TST+ groups, while the concentration of all the lipids (TC, TG, LDL-C and HDL-C) remained lower in the HIV−TB+ patients compared to HIV−TST− (*P* < 0.05 for all comparisons). Moreover, the CD4+ T cell count was significantly higher in HIV−TB+ after ATT compared to HIV−TB+ patients at baseline and normalized to the CD4+ T cell count of HIV−TST+ and/or HIV−TST+ at baseline (Table 4).

## 4. Discussion

This study was conducted to assess any difference in concentration of lipid profiles (TC, TG, LDL-C, and HDL-C) between TB patients with and without HIV infection and latent TB or healthy control individuals and to determine the level of lipid profiles in response to TB treatment.

At the baseline, our result shows the concentration of lipid profiles (TC, TG, LDL-C, and HDL-C) in HIV negative active TB patients was lower compared to both HIV negative latent TB and healthy controls, while those lipid parameters in latent TB groups were not significantly different from healthy groups.

Low TC, LDL-C, and HDL-C concentrations observed in Ethiopian active TB patients were in line with the studies conducted in Turkey [12], Egypt [16], and Benin [17]. In addition, low cholesterol concentration was observed in the homeless TB patients compared to nonhomeless TB patients and healthy men elsewhere [18]. The low concentration of lipid profiles in patients with pulmonary tuberculosis is correlated with disease severity and improper functioning of the immune system [6, 19]. Our findings showed that 73, 82, 94, and ≈88% of TB patients had low TC, TG, LDL, and HDL-C concentration levels, respectively, at the baseline compared to 4, 32, 68, and 36% of healthy controls. This alerts physicians to encourage regular lipid profile testing. Even though researchers recommend TC, HDL, and LDL concentration levels as biomarkers for pulmonary tuberculosis [20], these parameters are not yet used in Ethiopia. Therefore, our findings suggest the need to test lipid profiles in TB patients at the baseline for better patient management and treatment outcomes. Further research is also warranted to investigate the factors contributed to the lower lipid profile in TB patients as well as investigate the effect of the lower lipid profile in disease severity in TB patients.

We also showed low concentration of TC, LDL-C, and HDL-C in HIV+TB+ patients compared with HIV−TB+ and HIV+TST+ patients compared to HIV−TST+ groups. A study conducted in Nigeria shows the significantly reduced level of HDL-C and TC in HIV positive patients compared with HIV negative controls [21]. This is similar to our result which is lower concentration of HDL-C and TC in HIV+TB+ and HIV+TST+ compared to HIV−TB+ and HIV−TST+, respectively, which might be related to the main feature of AIDS, that is, depletion of CD4+ T cells and inhibiting other immune systems [22]. Active TB and TB-HIV coinfection pose an additional metabolic, physical, and nutritional burden, resulting in further increase in energy expenditure, malabsorption, and micronutrient deficiency [13]. In fact, the combination of malnutrition leading to decreased “supplementation” of lipid and reduction of immune parameters might be representing a pathogenic loop of TB. The Mtb activates invaded macrophages resulting in free radical burst. High serum levels of the free radicals and high concentration of lipid peroxidation products are characteristics of patients with advanced tuberculosis [17, 23, 24]. The peroxidation could cause reduced concentration of serum lipids and tissue inflammation [25]. The previous study demonstrated that the CD4+ T cell and/or FoxP3 gene expression had been correlation with both TC and LDL-C [26]. The increasing of lipid profiles was also stimulation of immune system to increase the white blood cell [27]. Another previous study confirmed that hypercholesterolemia induced an accumulation of regulatory T lymphocytes in the atherosclerotic aorta and spleens in mice, but the expression of functional selectin ligands was decreased [28].

We assessed also the level of lipids in response to ATT. We showed that the concentration level of TC, TG, LDL-C, and HDL-C was significantly increased in ATT treated TB patients at 6 months compared to baseline. The concentration of those lipid parameters in TB patients at the end of ATT treatment was similar compared to concentrations in latent TB infection, while still being significantly lower compared to healthy controls

The finding that TB patients with ATT significantly increased the lipid profile concentration compared to baseline is consistent with the previous findings [17, 28], while the concentration of the lipid profile level in TB patients at the end of ATT treatment was not normalized to the level of healthy controls. The increment of lipid profile concentration after taking ATT treatment in TB patients might be due to the nutritional status and immune function improing [29] and the cleaning of circulating of bacilli in the blood [30]. Our results also showed an increase in lipid concentration levels in ATT treated TB patients which might be related to increasing in CD4 cells from baseline to 6 months' follow-up.

Our findings should be interpreted within the context of the study and its limitations. We used a five-year stored plasma sample which had little effect (≈2% decrement for TC, TG, and HDL-C) of 7 years' stored plasma samples studied in United States of America [31, 32]. We did not collect individual-level dietary history and cannot comment on the role of dietary intake. We do not have data on regional body composition or blood pressure, so we were unable to assess the risk of metabolic syndrome or coronary heart disease or develop predictors for the same in this population; furthermore, we were unable to compare lipid levels with other well-known markers of inflammation (C-reactive protein (CRP) and albumin concentrations) because there is no data from the prospective study. Our follow-up end point at 6 months may not have been enough to assess long-term changes in lipids. The limited sample size may affect the adequate statistic power to test the study hypothesis.

In conclusion, according to our finding, we found that patients with pulmonary TB have low lipid profiles (TC, TG, LDL-C, and HDL-C) that indicate TB diseases might be a risk factor for low lipid profiles at diagnosis time point. These low lipid profiles appear to be correctable to normal levels of HIV−TST+ (but not HIV−TST−) after anti-TB treatment. Further research is needed with larger number of patients with individual-level dietary history and longer follow-up periods in order to provide additional support to this finding.

## Figures and Tables

**Table 1:** Baseline study population characteristics.

Characteristics	HIV+TB+ (*n* = 44)	HIV−TB+ (*N* = 49)	HIV+TST+ (*N* = 17)	HIV−TST+ (*N* = 24)	HIV−TST− (*N* = 25)	*P* value
*Demographic data*						
Median age, years	30.5 ± 16.2	39 ± 13.8	44 ± 12.9	41 ± 13.3	41 ± 12.9	0.0737
Female, *n* (%)	21 (48)	24 (49)	8 (47)	11 (46)	12 (48)	0.999
Median of BMI, kg/m2 (IQR)	26.5 (24–28)	27 (23.5–28)	27 (23–29)	27 (24.5–29)	28 (26–30)	0.0641
*Nutritional status*						
Severe malnutrition, *n* (%)	0 (0)	0 (0)	0 (0)	0 (0)	0 (0)	
Moderate malnutrition, *n* (%)	0 (0)	0 (0)	0 (0)	0 (0)	0 (0)	
Mild malnutrition, *n* (%)	0 (0)	0 (0)	0 (0)	0 (0)	0 (0)	
Normal, *n* (%)	13 (30)	16 (33)	7 (41)	6 (25)	4 (16)	
Overweight, *n* (%)	31 (70)	33 (67)	10 (59)	18 (75)	21 (84)	
*CD4+ T cell count*						
Median CD4+ count (IQR)	180 (112–239.5)	234 (146–345)	200 (146–267)	333 (234– 478)	345 (234–401)	**0.0001**
Median CD4+ T cell count ≤ 200, *n* (%)	24 (42)	19 (33)	6 (11)	3 (5)	5 (9)	**0.003**

Data indicate medians ± standard deviations unless stated otherwise. BMI cutoff of <18. Kg/m^2^ was used to define underweight. A CD4+ T cell count cutoff of <200 cells/*µ*l was used to define lymphocytopenia, *n* (%): number of patients (percentage of patients); BMI: Body Mass Index.

**Table:** (a) Baseline lipid profile levels (mg/dl) among HIV negative study groups (month 0)

Lipid name	HIV−TB+ (*n* = 49)	HIV−TST+ (*n* = 24)	HIV−TST− (*n* = 25)	*P* value		
				HIV−TB+ versus HIV−TST+ *P* value	HIV−TB+ versus HIV−TST− *P* value	HIV−TST+ versus HIV−TST− *P* value
TC	108 ( 87–130)	149 (140.5–162)	158 (150–192)	**<0.001**	**<0.001**	0.119
TG	75 (66–84)	83.5 (73–110.5)	119 (82–119)	**0.027**	**<0.001**	0.180
LDL	59.3 (46.9–73.2)	73.35 (63.8–87.2)	80.4 (73.5–105.1)	**0.001**	**<0.001**	0.072
HDL	26.5 (21.1–31.8)	42.4 (37.05–49.3)	44.7 (37.4–54.6)	**<0.001**	**<0.001**	0.276

Median (interquartile range) micronutrient level values are shown at baseline and significant differences between study groups were determined using Kruskal-Wallis H and Wilcoxon Mann–Whitney test.

**Table:** (b) The proportional of the abnormality of lipid profile level among study groups at baseline

Lipid name	HIV+TB+ (*n* = 44)	HIV−TB+ (*n* = 49)	HIV+TST+ (*n* = 17)	HIV−TST+ (*n* = 24)	HIV−TST− (*n* = 25)	*P* value using Pearson Chi^2^
TC < 130 mg/dl, N (%)	38 (86)	36 (73)	11 (65)	2 (8)	4 (4)	**<0.001**
TC ≥ 200 mg/dl, N (%)	0 (0)	0 (0)	0 (0)	1 (4)	6 (24)	**<0.001**
TG < 90 mg/dl, N (%)	30 (68)	40 (82)	13 (76)	14 (58)	8 (32)	**0.001**
TG ≥ 150 mg/dl, N (%)	0 (0)	0 (0)	0 (0)	2 (8)	2 (8)	0.056
LDL < 100 mg/dl, N (%)	43 (98)	46 (94)	15 (88)	19 (79)	17 (68)	**0.002**
LDL ≥ 130 mg/dl, N (%)	0 (0)	1 (2)	0 (0)	1 (4)	1 (4)	0.652
HDL < 40 mg/dl, N (%)	43 (98)	43 (88)	13 (76)	10 (42)	9 (36)	**<0.001**
HDL > 40 mg/dl, N (%)	1 (2)	6 (12)	4 (24)	14 (58)	16 (64)	**0.000**
TC < 130 & TG < 90 & LDL < 100 & HDL < 40	25 (40)	28 (44)	8 (13)	2 (3)	0 (0)	

**Table 3:** Lipid profile levels (mg/dl) of active TB and latent TB coinfected with HIV compared to without HIV infection.

Lipid name	HIV+TB+ (*n* = 40)	HIV−TB+ (*n* = 20)	^*∗*^ *P* value	HIV+TST+ (*n* = 20)	HIV−TST+ (*n* = 20)	^‡^ *P* value
TC	88 (71.5–112)	108 (87–130)	**0.012**	106 (83–140)	149 (140.5–162)	**<0.001**
TG	76 (68–94)	75 (66–84)	0.200	80 (67–89)	83.5 (73–110.5)	0.315
LDL-C	42 (32.45– 63.1)	59.3 (46.9–73.2)	**0.008**	54.2 (41–70.2)	73.35 (63.8–87.2)	**0.004**
HDL-C	17.65 (12.35–22.65)	26.5 (21.1–31.8)	**<0.001**	25.3 (18.1–37.6)	42.4 (37.05– 49.3)	**<0.001**

Median (interquartile range) of lipid profile level values at baseline is shown. Comparison among the groups was determined using Wilcoxon Mann–Whitney test; ^*∗*^*P* value for the comparison between active TB groups with and without HIV; ^‡^*P* value for the comparison between latent TB groups with and without HIV.

**Table 4:** ATT response on lipid profile concentration in HIV−TB+ patients after treatment.

Lipid profile name	HIV−TB+ at M0	HIV−TB+ at M6	HIV−TST+	HIV−TST−	HIV−TB+ (M6) versus HIV−TB+ (M0) *P* value	HIV−TB+ (M6) versus HIV−TST+ (M0) *P* value	HIV+TB+ (M6) versus HIV−TST− (M0) *P* value
TC	108 ( 87–130)	137 (122–166)	149 (140.5–162)	158 (150–192)	0.0000	0.8638	0.0047
TG	75 (66–84)	76 (65–87)	83.5 (73–110.5)	119 (82–119)	0.4212	0.1046	0.0007
LDL-C	59.3 (46.9–73.2)	71.2 (56.8–92.4)	73.35 (63.8–87.2)	80.4 (73.5–105.1)	0.0120	0.7253	0.0110
HDL-C	26.5 (21.1–31.8)	35.9 (32.4–45.1)	42.4 (37.05– 49.3)	44.7 (37.4–54.6)	0.0000	0.9466	0.0027
CD4+	234 (146–345)	345 (234–566)	333 (234–478)	345 (234–401)	0.0063	0.4321	0.3865

Median (interquartile range) of lipid profile concentration and CD4+ T-cell count values are shown. Significant differences between HIV−TB+ patients at M0 and M6 were determined using Wilcoxon signed-rank test. Significant differences between HIV−TB+ patients at M6 and HIV−TST+ or HIV−TST− at M0 were determined using Wilcoxon Mann–Whitney test.
